# *Rnf20* deficiency in adipocyte impairs adipose tissue development and thermogenesis

**DOI:** 10.1007/s13238-020-00770-2

**Published:** 2020-08-14

**Authors:** Xiaojuan Liang, Cong Tao, Jianfei Pan, Lilan Zhang, Lulu Liu, Ying Zhao, Yiping Fan, Chunwei Cao, Jiali Liu, Jin Zhang, Sin Man Lam, Guanghou Shui, Wanzhu Jin, Wei Li, Jianguo Zhao, Kui Li, Yanfang Wang

**Affiliations:** 1 State Key Laboratory of Animal Nutrition, Institute of Animal Science, Chinese Academy of Agricultural Sciences, Beijing, 100193 China; 2 Department of Animal Science, China Agricultural University, Beijing, 100193 China; 3 Institute of Zoology, Chinese Academy of Sciences, Beijing, 100101 China; 4 College of Biological, Chemical Sciences and Engineering, Jiaxing University, Jiaxing, 314001 China; 5 Institute of Genetics and Developmental Biology, Chinese Academy of Sciences, Beijing, 100101 China; 6 Savaid Medical School, University of Chinese Academy of Sciences, Beijing, 100049 China; 7 College of Life Sciences, Qingdao Agricultural University, Qingdao, 266109 China

**Keywords:** RNF20, fat loss, adipose tissue development, thermogenesis

## Abstract

RNF20, an E3 ligase critical for monoubiquitination of histone H2B at lysine 120 (H2Bub), has been implicated in the regulation of various cellar processes; however, its physiological roles in adipocytes remain poorly characterized. Here, we report that the adipocyte-specific knockout of *Rnf20* (ASKO) in mice led to progressive fat loss, organomegaly and hyperinsulinemia. Despite signs of hyperinsulinemia, normal insulin sensitivity and improved glucose tolerance were observed in the young and aged CD-fed ASKO mice. In addition, high-fat diet-fed ASKO mice developed severe liver steatosis. Moreover, we observed that the ASKO mice were extremely sensitive to a cold environment due to decreased expression levels of brown adipose tissue (BAT) selective genes, including uncoupling protein 1 (*Ucp1*), and impaired mitochondrial functions. Significantly decreased levels of peroxisome proliferator-activated receptor gamma (*Pparγ*) were observed in the gonadal white adipose tissues (gWAT) from the ASKO mice, suggesting that *Rnf20* regulates adipogenesis, at least in part, through *Pparγ*. Rosiglitazone-treated ASKO mice exhibited increased fat mass compared to that of the non-treated ASKO mice. Collectively, our results illustrate the critical role of RNF20 in control of white and brown adipose tissue development and physiological function.

**Electronic supplementary material:**

The online version of this article (10.1007/s13238-020-00770-2) contains supplementary material, which is available to authorized users.

## Introduction

Obesity has become a major health concern and an important worldwide social problem. Obesity occurs when energy intake increases or energy expenditure decreases (Tseng et al., 2010) and is a significant risk factor for a vast number of metabolic diseases, such as cardiovascular disease, type 2 diabetes, hypertension, fatty liver disease and atherosclerosis (Jin and Patti, 2009). While restriction of caloric intake is an effective way to defend against obesity, modifications to metabolic efficiency and increased energy expenditure in adipose tissues have been identified as alternative strategies to fight obesity and prevent related metabolic diseases.

Three major types of adipocytes have been identified in most mammals. White adipose tissue (WAT) is a major organ that stores excess energy as triglycerides and releases free fatty acids (FFAs) to meet energy demands. In contrast, brown adipose tissue (BAT), which has numerous mitochondria and is specialized to dissipate energy in the form of heat, has been identified in rodents and newborn humans to function as a defense mechanism against hypothermia (Oelkrug et al., 2015). Recently, BAT was also found in the lower neck and supraclavicular areas in adult human by 18F-fluorodeoxyglucose positron emission tomography-computed tomography (18F-FDG-PET/CT) scanning (Van Marken Lichtenbelt et al., 2009; Virtanen et al., 2009; Zingaretti et al., 2009). Another thermogenic adipocyte, named beige or “brite” (brown in white) adipocyte, was recently identified in WAT exposed to various environmental stimuli, such as cold exposure (Liang et al., 2019), exercise (Phillips, 2019), or treatment with β-adrenergic receptor agonist (Himms-Hagen et al., 2000). Accumulating evidence has shown that activating BAT and promoting the browning of WAT burns fat and is applicable for the treatment of obesity and related metabolic diseases.

As a histone posttranslational modification (PTM), monoubiquitination at lysines on histones H2A or H2B, has been reported to influence the nature of the chromatin landscape (Kouzarides, 2007; Dawson et al., 2012; Xu et al., 2016). It has been demonstrated that lysine 120 at which monoubiquitination of histone H2B occurs is the only site of histone monoubiquitination shown to result in the physical disruption of chromatin strands, those leading to open and accessible fiber conformations (Minsky et al., 2008; Fierz et al., 2011). In contrast to polyubiquitination, which marks a protein for proteasomal degradation, histone H2B lysine 120 monoubiquitination (hereafter referred to as H2Bub) has been reported to play a vital role in some of the most fundamental biological processes at both the molecular and cellular levels (Fuchs and Oren, 2014). It is therefore not surprising that global knockout of RNF20, an E3 ligase that catalyzes the monoubiquitination of histone H2B at lysine 120, results in very early embryonic lethality (Fuchs and Oren, 2014).

RNF20 has been reported to be essential for the transcriptional regulation of gene expression (Wu et al., 2020), double-strand break repair (So et al., 2019) and homologous recombination (Nakamura et al., 2011), and it plays critical roles in various cellular processes, including cell differentiation (Fuchs et al., 2012; Karpiuk et al., 2012; Vethantham et al., 2012; Liang et al., 2018) and apoptosis (Woo Park et al., 2015). In addition, RNF20 is also involved in regulating specific physiological process, such as tumorigenesis (Duan et al., 2016; Tarcic et al., 2016; Sethi et al., 2018) and spermatogenesis (Xu et al., 2016). Its role in lipogenesis has also been studied. Lee et al. demonstrated the role of RNF20 in hepatic fatty acid metabolism by showing that it regulates the activity of sterol regulatory element binding protein1c (SREBP1c), the key transcription factor for *de novo* lipogenesis, and the lipogenic genes that are targets of SREBP1c (Lee et al., 2014). Very recently, the same group revealed that *Rnf20*^+/−^ mice are lean on a chow diet, and these mutants exhibited alleviated systemic insulin resistance, reduced expansion of fat tissue even when fed a high-fat diet. Mechanistically, RNF20 plays a role by promoting proteasomal degradation of nuclear corepressor 1 (NCoR1), which leads to the stimulation of the transcriptional activity of PPARγ (Jeon et al., 2020). However, the contribution of RNF20 defects in other metabolic tissues cannot be excluded in *Rnf20*^+/−^ mice, and the importance of adipocyte RNF20 in fat deposition and fat-tissue-mediated metabolic homeostasis remains unknown.

In this study, we created a conditional ablation of *Rnf20* in adipose tissues to critically examine the requirement for *Rnf20* in fat accumulation and body composition. The changes in lipid metabolism, glucose and energy homeostasis due to its specific action in fat tissues were also explored.

## Results

### Adipocyte-specific knockout of *Rnf20* in mice leads to progressive fat loss

RNF20 expression was examined in various tissues from wild-type C57BL/6J mice, and the immunoblotting and quantitative data showed that RNF20 was ubiquitously expressed (Fig. S1A and S1B). To examine the role of *Rnf20* in mature adipocytes, we generated mice in which *Rnf20* was specifically depleted in adipose tissues. Homozygous *Rnf20*^*Flox*/*Flox*^ mice were obtained as described previously (Xu et al., 2016) and crossed with transgenic mice expressing Cre recombinase under the control of the *adiponectin* gene promotor (*adipoq-Cre*). The resulting *Rnf20*^*Flox*/+^*Cre*^+^ progeny were then crossed with *Rnf20*^*Flox*/*Flox*^ mice to generate the *Rnf20*^*Flox*/*Flox*^Cre^+^ mice (hereafter referred to as ASKO mice). The litter mates of the *Rnf20*^*Flox*/*Flox*^Cre^+/−^ mice were used as controls and are referred to as wild-type (WT) mice. The mRNA levels of *Rnf20* gene were dramatically reduced in adipose tissues, including sWAT, gWAT and BAT, in the 2-month-old ASKO mice (Fig. S1C). Immunoblotting and quantitative analysis showed the significant loss of RNF20 protein in adipose tissues of the ASKO mice and a weak band in the ASKO mice suggested that RNF20 was not deleted in other cells, such as vascular cells and macrophages (Fig. S1D and S1E). The expression levels of RNF20 in other peripheral organs were not altered (Fig. S1F). These results demonstrated that we had successfully generated adipocyte-specific *Rnf20* knockout mice.

**Figure 1:**
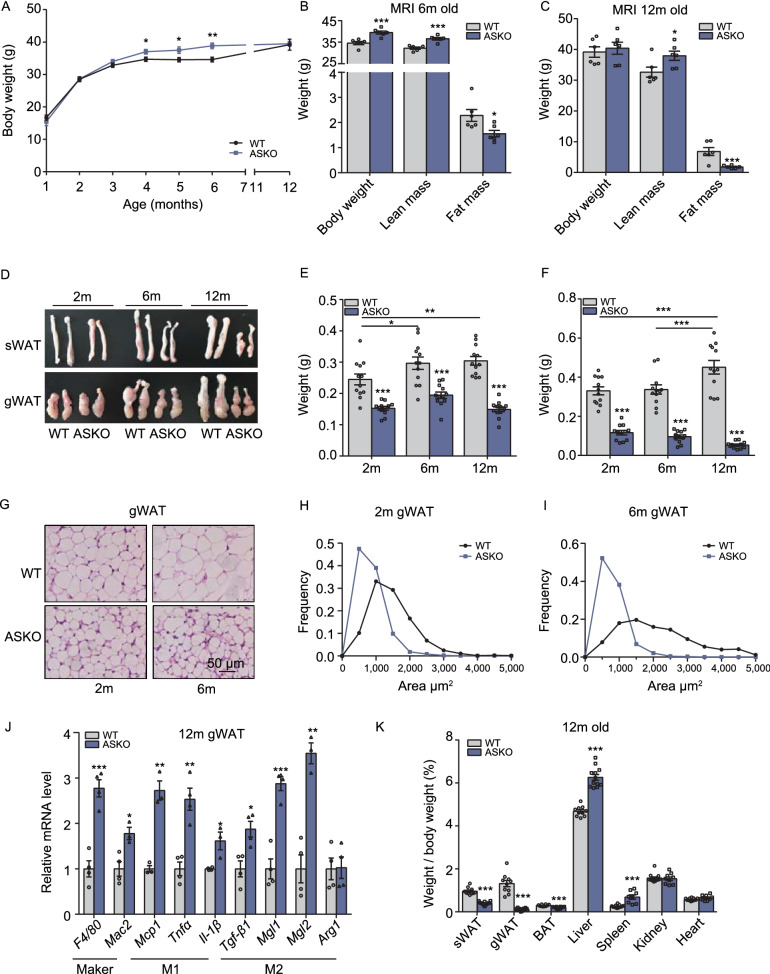
**Adipocyte-specific knockout of** ***Rnf20*** **in mice leads to progressive fat loss**. (A) Body weight of the male WT and ASKO mice fed a chow diet (*n* = 8–10). (B and C) MRI analysis of the body compositions of the 6- (B) and 12 month-old (C) WT and ASKO mice (*n* = 6). (D) Gross morphology of sWAT and gWAT from 2-, 6- and 12-month-old WT and ASKO mice. (E and F) Weight of sWAT (E) and gWAT (F) from 2-, 6- and 12-month-old WT and ASKO mice (*n* = 8). Note that the testis was dissected together with the gWAT depots.(G) H&E staining of gWAT sections derived from WT and ASKO mice at 2 and 6 months of age. Scale bars: 50 μm. (H and I) The distribution of adipocyte size of gWAT from 2- and 6-month-old WT and ASKO mice.(J) Relative mRNA levels of macrophage markers and inflammatory cytokines in gWAT from 12-month-old WT and ASKO mice (*n* = 4). (K) Relative organ weights of the 12-month-old mice (*n* = 10). All quantitative data are presented as the means ±s.e.m. *P < 0.05, **P < 0.01 and ***P < 0.001 for differences between WT and ASKO mice

To determine whether knocking out RNF20 affected the body composition, both the WT and ASKO mice were fed a chow diet, and their body weights were monitored for 12 months. Significantly heavier body weight was observed in the ASKO mice older than 4 months (Fig. 1A), and this observation was confirmed by evidence from a magnetic resonance imaging (MRI) analysis of 6-month-old mice (Fig. 1B). Although the body weight of the ASKO mice was slightly increased, the MRI data revealed significantly increased lean mass and decreased fat mass in 6- and 12-month-old mice (Fig. 1B and 1C). The subcutaneous WAT (sWAT) and gonadal WAT (gWAT) were harvested from both the WT and ASKO mice at different ages, and a representative photo of gross morphology is shown in Fig. 1D. It is noteworthy that the weight of sWAT in the ASKO mice was all significantly lower than those from WT mice at different ages (Fig. 1E). Particularly, the weight of gWAT from the ASKO mice exhibited significant decreases with age, while those from WT mice gradually increased (Fig. 1F). Histological analysis of gWAT from 2- and 6-month-old mice showed that the white adipocytes of ASKO mice were much smaller than those from WT mice (Fig. 1G–I). However, the weights of other peripheral organs, such as the liver and spleen, were found to be increased significantly in 12-month-old ASKO mice (Fig. 1K). Since crown-like structures in gWAT of ASKO were observed, the markers of inflammation and macrophage infiltration were evaluated. Our data showed the significant upregulation of these marker genes in gWAT from ASKO mice at different ages (Figs. 1J, S2A and S2B). Taken together, our results illustrate that the adipocyte-specific knockout of *Rnf20* resulted in progressive fat loss.

### Impaired lipogenesis and lipolysis in the gWAT of the ASKO mice

**Figure 2:**
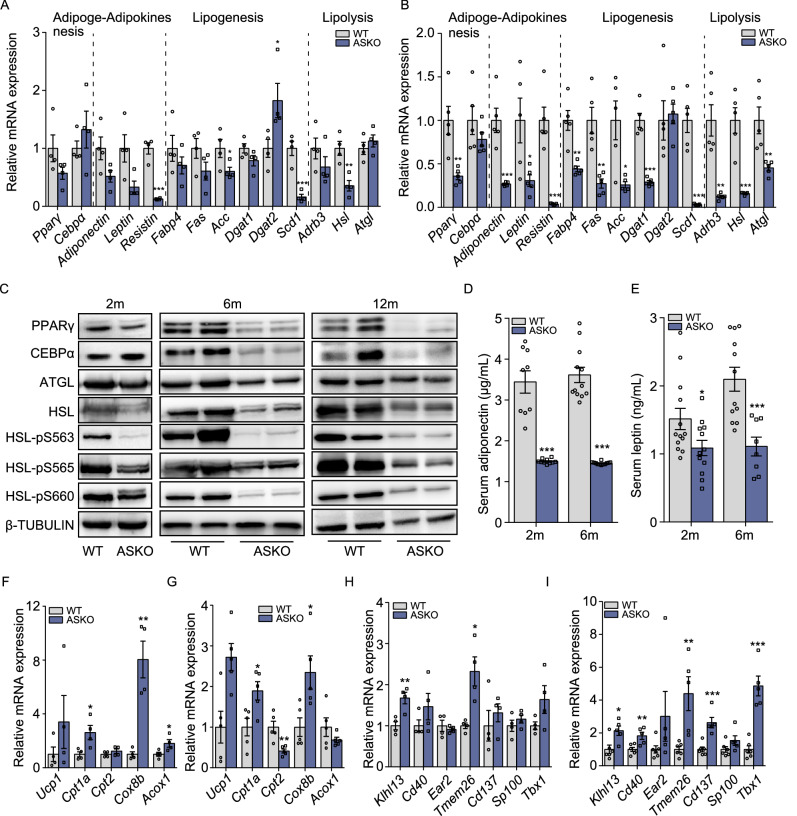
**Adipocyte-specific depletion of** ***Rnf20*** **suppresses the expression of lipogenesis and lipolysis related genes in the gWAT of mice**. (A and B) Expression levels of adipokine genes and genes related to adipogenesis, lipogenesis and lipolysis in the gWAT from the 2- (A) and 6-month-old (B) WT and ASKO mice (*n* = 6). (C) Western blots for lipogenesis and lipolysis markers in the gWAT from the 2-, 6- and 12-month-old WT and ASKO mice. (D and E) Serum adiponectin (D) and leptin (E) were measured in 2- and 6-month-old ASKO and WT mice (*n* = 10). (F and G) Expression levels of the *Ucp1* and fatty acids oxidation related genes in the gWAT from 2- (F) and 6-month-old (G) ASKO and WT mice (*n* = 6), respectively. (H and I) Expression levels of the beige formation related genes in the gWAT from 2- (H) and 6-month-old (I) ASKO and WT mice (*n* = 6), respectively. All quantitative data are presented as the means ± s.e.m. *P < 0.05, **P < 0.01 and ***P < 0.001 for differences between the WT and ASKO mice

To investigate the potential mechanism of the dramatically reduced WAT mass, we examined the expression levels of genes involved in adipogenesis (*Pparγ* and *Cebpα*), genes encoding adipokines (*Adiponectin*, *Leptin* and *Resistin*), lipogenesis (*Fabp4*, *Fas*, *Acc*, *Dgat1*, *Dgat2* and *Scd1*) and lipolysis (*Adrb3*, *Hsl* and *Atgl*) in gWAT from both the WT and ASKO mice at 2 or 6 months old age. Our results showed that most of these genes were decreased in the gWAT from 2-month-old ASKO mice (Fig. 2A), and the decrease was significant in the 6-month-old ASKO mice (Fig. 2B). The downregulation of *Pparγ*, *Cebpα* and lipolysis genes, including *Hsl* and *Atgl*, were confirmed by immunoblot analysis (Fig. 2C). Serum adiponectin and leptin were also found to be significantly downregulated in the 2- and 6-month-old ASKO mice, as ascertained by ELISAs (Fig. 2D and 2E).

In addition, we measured the expression levels of *Ucp1* and fatty acid oxidation related genes, including, *Ucp1*, *Cpt1a*, *Cpt2*, *Cox8b* and *Acox1*, in the gWAT of both the WT and ASKO mice at their different ages. We found that most of these genes were significantly increased in gWAT from 2- and 6-month-old ASKO mice (Fig. 2F and 2G). Since the smaller adipocytes were observed in gWAT from ASKO mice, it is of interest to study whether the known beige selective genes, including *Klhl13*, *Cd40*, *Tmem26*, *Cd137*, *Sp100*, *Tbx1* and *Slc27a1*, were changed in gWAT of ASKO mice. The results showed that only *Klhl13* and *Tmem26* were upregulated in the gWAT of 2-month-old ASKO mice (Fig. 2H), while most of them were significantly induced in 6-month-old ASKO mice (Fig. 2I).

### ASKO mice show hyperinsulinemia but protects against age-associated glucose intolerance

**Figure 3:**
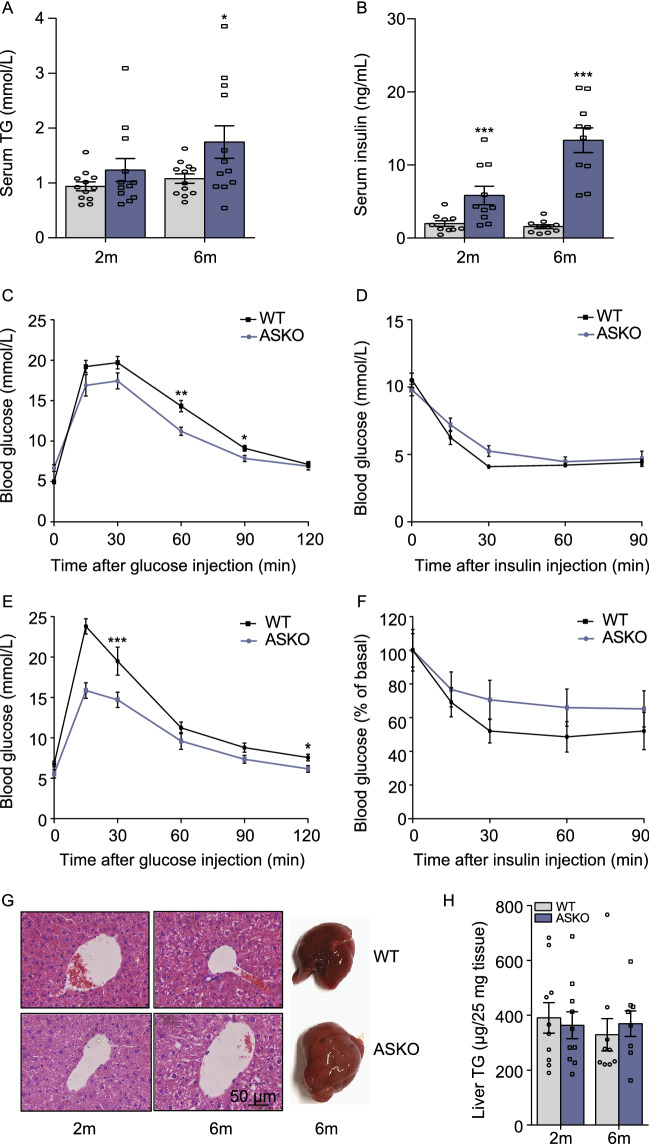
**ASKO mice are protected against age-induced glucose intolerance**. (A and B) Levels of triacylglycerol (A) and insulin (B) in the serum of 2- and 6-month-old WT and ASKO mice (*n* = 10). (C and E) Glucose tolerance tests were performed with 2- (C) and 12-month-old (E) WT and ASKO mice (*n* = 10). (D and F) Insulin tolerance test were performed with 2- (D) and 12-month-old (F) WT and ASKO mice (*n* = 10). (G) H&E staining of liver sections from 2- and 6-month-old WT and ASKO mice, and the representative image of liver from 6-month-old WT and ASKO mice. (H) Hepatic triacylglycerol contents in the WT and ASKO mice at different ages (*n* = 10). The results are presented as the means ± s.e.m. *P < 0.05, **P < 0.01 and ***P < 0.001 for differences between the WT and ASKO mice

Because defective adipose lipid storage often results in increased circulating triglyceride levels and ectopic lipid deposition, we examined the levels of TGs and insulin in serum from both groups of mice at different ages. Our data showed that the 6-month-old ASKO mice exhibited slight but significantly increased levels of serum TGs (Fig. 3A) and insulin (Fig. 3B). Severe fat loss often leads to metabolic disturbances, such as glucose intolerance and insulin resistance in humans and mice. Surprisingly, the glucose tolerance test showed that the 2- and 6-month-old ASKO mice had improved glucose sensitivity (Fig. 3C and 3E). In contrast, the insulin tolerance test showed that the ASKO mice had slight decreased insulin sensitivity, but this difference did not reach statistical significance (Fig. 3D and 3F).

As described above, the weights of the livers in the ASKO mice were significantly higher than those from the WT mice (Fig. 1K). The representative image of liver from 6-month-old WT and ASKO mice were shown in Fig. 3G. To detect whether fat loss in ASKO mice leads to fatty liver, the livers of WT and ASKO mice were subjected to H&E staining. Histological analysis revealed that the 2- and 6-month-old ASKO mice did not develop the severe fatty liver, compared to those from WT mice (Fig. 3G). In addition, the hepatic TGs levels were not significantly changed in both 2- and 6-month-old ASKO mice (Fig. 3H).

### ASKO mice are susceptible to HFD-induced fatty liver

**Figure 4:**
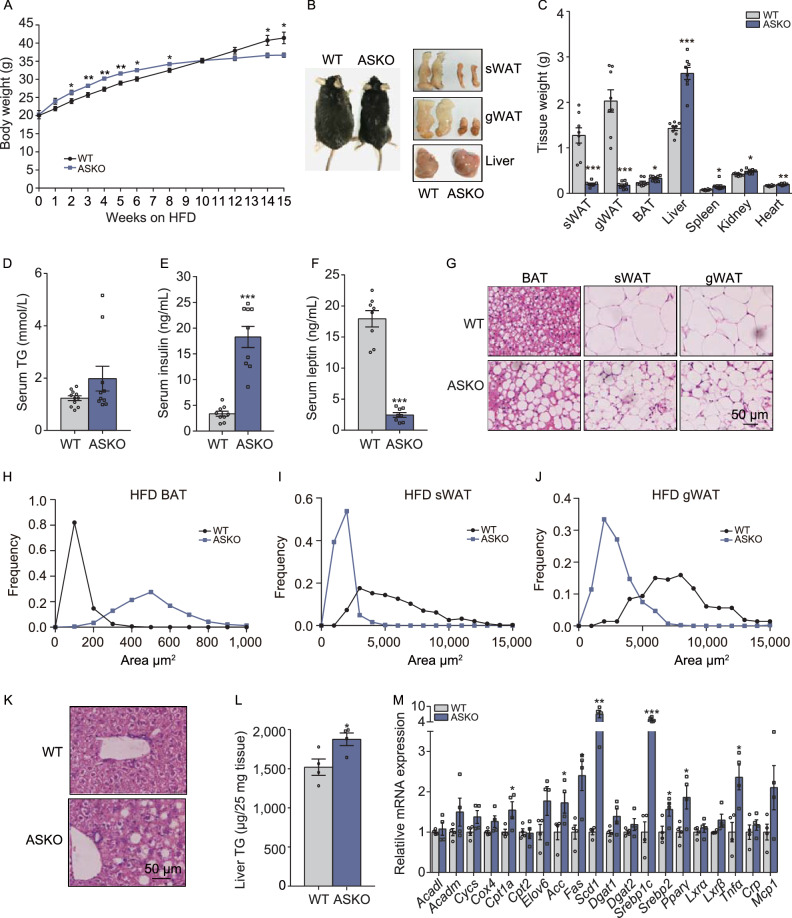
**ASKO mice are susceptible to HFD-induced fatty liver**. (A) Body weight of the WT and ASKO mice fed the HFD for 15 weeks (*n* = 8). (B) Representative photographs of mice after HFD treatment and the photographs of the sWAT, gWAT, and liver from HFD-fed WT and ASKO mice. (C) Tissue weights of the HFD-fed mice (*n* = 8). (D–F) Levels of TG (D) insulin (E) and leptin (F) in the serum of both HFD-fed WT and ASKO mice (*n* = 8). (G) H&E staining of the sWAT, gWAT and BAT from the HFD-fed WT and ASKO mice. (H, I and J) The distribution of adipocyte size of BAT (H), sWAT (I) and gWAT(J) from HFD-fed WT and ASKO mice. (K) H&E staining of the liver from the HFD-fed WT and ASKO mice. Scale bars, 50 μm. (L) Levels of liver TG from both HFD-fed WT and ASKO mice (*n* = 8). (M) Expression levels of the genes related to lipogenesis,  fatty acids β-oxidation and inflammation in the livers in HFD-fed WT and ASKO mice (*n* = 6). Data are presented as the mean ± s.e.m. *P < 0.05, **P < 0.01,***P < 0.001

We studied the metabolic effects of adipocyte *Rnf20* deletion during continuous feeding of a HFD. The WT and ASKO mice were fed a 60% HFD for 15 weeks when their body weight reached 20 g. Clearly, after 10 weeks of HFD, the body weights of the ASKO mice were greater than those of the WT mice. After 15 weeks on HFD, the WT mice gained significantly more body weight than the ASKO mice (Fig. 4A). Representative photos of the WT and ASKO mice ant their adipose tissues were shown in Fig. 4B. Furthermore, we found that the weights of the fat pads of the HFD-fed ASKO mice were significantly lower than those of the control mice (Fig. 4B and 4C). However, the weights of the peripheral organs, namely, the heart, liver, kidney and spleen, were observed to be increased significantly in the ASKO mice compared with those from the WT mice (Fig. 4C). The HFD-fed ASKO mice exhibited significantly higher levels of serum TGs (Fig. 4D) and insulin (Fig. 4E) and significantly lower levels of leptin (Fig. 4F). Histological analysis revealed that the WAT adipocytes of the ASKO mice were much smaller than those of the WT mice (Fig. 4G, 4I and 4J), while the lipid droplets in the brown adipocytes were markedly larger, indicating a profound whitening of interscapular BAT (Fig. 4G and 4H). In addition, the HFD-fed ASKO mice exhibited a severe fatty liver phenotype, as revealed by gross morphology analysis (Fig. 4B), H&E staining (Fig. 4K), and hepatic triglyceride quantification (Fig. 4L). Furthermore, we found that the genes involved in hepatic *de novo* lipogenesis were induced in the liver of the HFD-fed ASKO mice, as the differences in *Acc*, *Scd1*, *Srebp1c*, *Srebp2* and *Pparγ* mRNA levels reached statistical significance (Fig. 4M). Fatty acid oxidation*-*related genes were not changed, except for *Cpt1a* (Fig. 4M).

### The BAT program is impaired in ASKO mice

**Figure 5:**
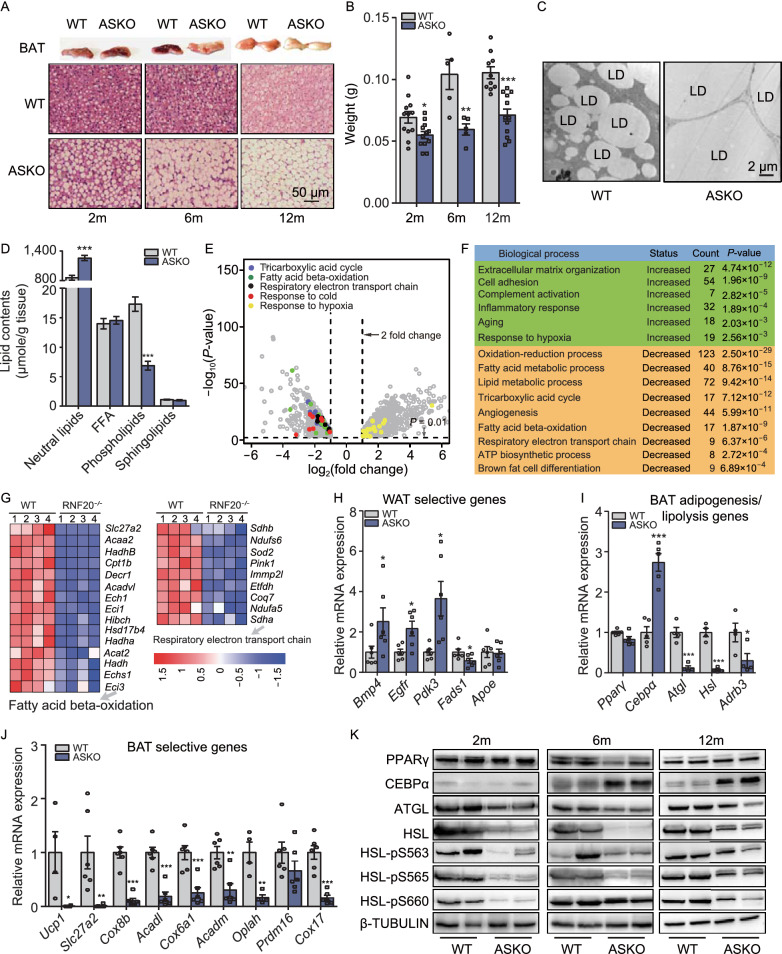
**The brown program is impaired in ASKO mice**. (A) Gross morphology and H&E staining of the BAT in the 2-, 6-, and 12-month-old WT and ASKO mice. (B) Weight of BAT from the 2-, 6-, and 12-month-old WT and ASKO mice. (C) TEM images of the BAT from the 6-month-old WT and ASKO mice. (D) Lipid profiles of four lipid classes in the BAT from the 6-month-old WT and ASKO mice (*n* = 5). (E) Volcano plot of the genes displaying a significant difference in the expression in the BAT from the 6-month-old WT and ASKO mice (*n* = 4). Colored dots indicate genes in the enriched gene ontology terms. (F) The significantly enriched GO annotations in BAT from the ASKO mice compared with those from the WT mice. (G) The heatmaps were built with the differentially expressed genes which were enriched in GO annotations of fatty acid β-oxidation and respiratory electron transport chain. (H–J) mRNA expression levels of WAT selective genes (H), lipogenesis and lipolysis related genes (I) and BAT selective genes (J) in the BAT from the 12-month-old WT and ASKO mice (*n* = 6). (K) Western blots for lipogenesis and lipolysis markers in the BAT from 2-, 6-, and 12-month-old WT and ASKO mice. Data are presented as the mean ± s.e.m. *P < 0.05, **P < 0.01, ***P < 0.001

The BAT in the ASKO mice at 2, 6 and 12 months of age displayed profound morphological whitening (Fig. 5A) with the significant decrease of tissue mass (Fig. 5B). The whitening of the BAT was confirmed by the switch from a multilocular to a unilocular morphology, as determined from the results of H&E staining (Fig. 5A) and TEM scanning (Fig. 5C). To identify the lipid components of the BAT in ASKO mice, the lipidomic analysis of BAT from both WT and ASKO mice at their 6 months old age was performed. The results revealed that the levels of neutral lipids were significantly upregulated, while those of phospholipids were significantly decreased in the BAT from ASKO mice (Fig. 5D). To better characterize the molecular signature of the BAT from ASKO mice, genome-wide RNA-sequencing of BAT from 6-month-old mice was performed, and a global overview of the changes in gene expression is shown in volcano plots (Fig. 5E). A total of 2,354 differentially expressed genes, which were screened based on the criteria of *P* < 0.05 and a fold change >2, were used for a Gene Ontology (GO) analysis. Notably, the GO terms for the tricarboxylic acid cycle, fatty acid β-oxidation, respiratory electron transport chain and response to cold were significantly enriched in the downregulated genes, as highlighted in the volcano plots (Fig. 5E and 5F). Significant changes in fatty acid β-oxidation and respiratory electron transport chain-related genes are profiled in the heatmap (Fig. 5G). Consistently, *Bmp4* and *Pdk3*, known WAT marker genes, were significantly upregulated (Fig. 5H), while most BAT marker genes, including *Ucp1*, *Slc27a2*, and *Cox8b*, were dramatically downregulated (Fig. 5J) in the BAT of the 12-month-old ASKO mice. Interestingly, opposite with the observation of significantly suppressed *Pparγ* and *Cebpα* in the gWAT of the ASKO mice (Fig. 2B and 2C), we found the unchanged *Pparγ* and the significantly induced *Cebpα* in the BAT of the ASKO mice (Fig. 5I and 5K). The lipolysis-related genes were downregulated in the BAT of the ASKO mice at both the mRNA (Fig. 5I) and protein levels (Fig. 5K). Taken together, our data demonstrated that the BAT program was impaired in ASKO mice.

### ASKO mice are extremely cold sensitive and develop hypothermia

**Figure 6:**
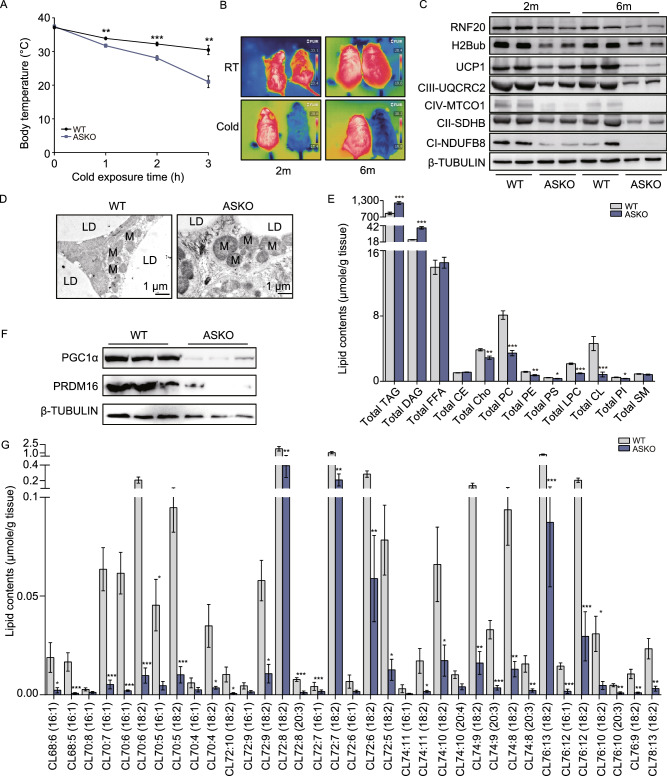
**ASKO mice are extremely cold sensitive and develop hypothermia**. (A) Rectal temperatures of the 6-month-old WT and ASKO mice which were recorded during the cold stimulation for 3 h (*n* = 8). (B) Representative infrared pictures of the 2- and 6-month-old mice were collected at the end of 3 h of cold exposure. (C) Representative western blot showing the levels of RNF20, H2Bub, UCP1 and OXPHOS proteins in the BAT from the 2- or 6-month-old WT and ASKO mice. (D) TEM images of the BAT from 6-month-old WT and ASKO mice. (E) Lipid profile of 12 lipid species in the BAT from the 6-month-old WT and ASKO mice (*n* = 5). (F) Representative western blot of PGC1α and PRDM16 in BAT of the 6-month-old WT and ASKO mice. (G) The cardiolipins, the major components of the mitochondrial cristae, were significantly decreased in the BAT of the mutant mice (*n* = 5). Data are presented as the mean ± s.e.m. *P < 0.05, **P < 0.01, ***P < 0.001

Since the depletion of RNF20 in the BAT led to dramatically decreased BAT marker genes (Fig. 5J), it was of interest to test whether BAT-mediated adaptive thermogenesis was affected in ASKO mice exposed to cold environment. The time-course cold challenge experiment was conducted with 6-month-old WT and ASKO mice and the rectal temperatures were monitored. As shown in Fig. 6A, the body temperatures of the ASKO mice were significantly lower than those of the WT mice at all time points tested (Fig. 6A). In addition, ventral infrared thermal images of both WT and ASKO mice at 2 and 6 months old age were collected at 3 h after cold exposure. Clearly, the ASKO mice are sensitive to the cold environment and developed hypothermia, as they exhibit the dramatic decreased surface temperatures (Fig. 6B). The expression levels of the UCP1 and OXPHOS protein were decreased in BAT from the 2- and 6-month-old ASKO mice (Fig. 6C). Moreover, TEM analysis of BAT from both WT and ASKO mice revealed an obvious decrease in the mitochondrial cristae density in the BAT of ASKO mice (Fig. 6D). The expression levels of two critical transcriptional factors in BAT biogenesis, including PGC1α and PRDM16, were found to be downregulated in BAT from 6-month-old ASKO mice (Fig. 6F). Furthermore, we found that most cardiolipins, which are the major component of mitochondrial cristae, were significantly decreased in the BAT of the ASKO mice (Fig. 6E and 6G). Taken together, these data suggested that BAT-mediated adaptive thermogenesis was impaired in ASKO mice partly due to mitochondrial dysfunction. Despite the impaired BAT program, the rate of O_2_ consumption was not significantly affected in the 6-month-old ASKO mice (Fig. S3). Moreover, the ASKO mice exhibited the higher physical activity, however, the difference didn’t reach the statistical significance (Fig. S4).

### RNF20 is required for adipocyte differentiation

**Figure 7:**
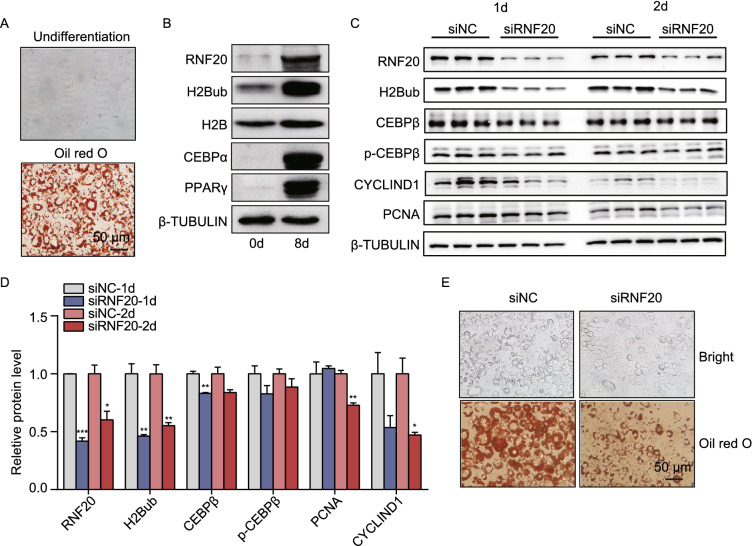
**RNF20 is required for adipocyte differentiation**. (A) 3T3-L1 cells were differentiated into mature adipocytes, as evidenced by the oil red O staining. (B) The expression levels of proteins, including RNF20, H2Bub, H2B and the two adipogenesis markers, in undifferentiated and differentiated adipocytes. (C) The expression levels of proteins involved in early adipogenesis (CEBPβ and p-CEBPβ) and proliferation (CYCLIND1 and PCNA) in siNC and siRNF20 transfected 3T3-L1 cells after 1 day and 2 days induction. (D) Data quantification of panel (C). (E) The efficiency of adipocytes differentiation was dramatically decreased in the siRNF20 cells based on lipid droplet formation under a bright field of microscopy and oil red O staining.

To investigate whether RNF20 plays a role in adipocyte differentiation, we examined its expression level in differentiated 3T3-L1 cells, a well-recognized mouse preadipocyte cell line. Successful *in vitro* differentiation was confirmed by oil red O staining (Fig. 7A) and the upregulation of adipocyte differentiation markers, PPARγ and CCAAT/enhancer-binding protein alpha (CEBPα) (Fig. 7B). Results from the immunoblot showed a dramatic increase in RNF20 and H2Bub in the differentiated adipocytes (Fig. 7B). Next, two siRNAs targeting *Rnf20* were designed, and their knockdown efficiency was measured. The results showed that both siRNAs could efficiently reduce the expression level of *Rnf20* (Fig. S5), and siRNF20-1 was used for the subsequent differentiation study. Furthermore, to examine the effect of RNF20 on early adipogenesis, the siNC or siRNF20 transfected 3T3-L1 cells were differentiated and harvested at 1 day and 2 days after induction. The expression levels of proteins that involved in the early adipogenesis (p-CEBPβ and CEBPβ) and proliferation (CYCLIND1, PCNA) were measured. The decreased protein levels were observed in siRNF20 cells (Fig. 7C and 7D), suggesting the critical regulatory roles of RNF20 in adipocyte proliferation and early adipocyte differentiation. In addition, the compromised differentiation efficiency was established in the fully differentiated siRNF20 cells (8 days after induction) based on lipid droplet formation under a bright field of microscopy and oil red O staining (Fig. 7E).

### Fat loss in the ASKO mice can be partially rescued by rosiglitazone treatment

**Figure 8:**
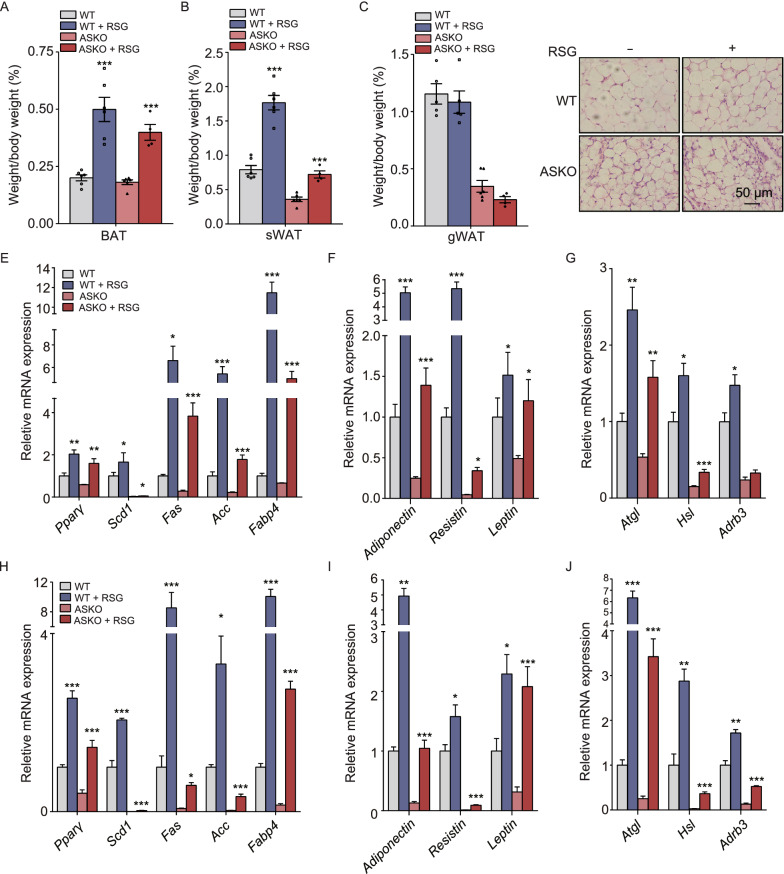
**Fat loss in the ASKO mice can be partially rescued by rosiglitazone treatment**. Three-month-old WT and ASKO mice were treated with or without rosiglitazone for 10 weeks. (A) BAT mass (B) sWAT mass and (C) gWAT mass in both WT and ASKO mice treated with or without rosiglitazone. (D) Histological analysis of the sWAT from both mice with or without rosiglitazone treatment (*n* = 6). (E–G) Expression levels of genes related to lipogenesis (E), adipokine (F) and lipolysis (G) in the gWAT from both mice treated with or without rosiglitazone (*n* = 6). (H–J) Expression levels of genes related to lipogenesis (H), adipokine (I) and lipolysis (J) in the sWAT from both mice treated with or without rosiglitazone (*n* = 6). Data are presented as the mean ± s.e.m. *P < 0.05, **P < 0.01, ***P < 0.001.

Because decreased levels of PPARγ were observed in WAT of ASKO mice, it was of interest to determine whether treatment with the PPARγ agonist, rosiglitazone, could rescue the fat loss in ASKO mice. Both WT and ASKO mice (3 months old) were treated with or without rosiglitazone for 10 weeks. Our data shows that rosiglitazone treatment resulted in an increase in BAT mass in both the WT and ASKO mice (Fig. 8A). The significant loss of sWAT in the untreated ASKO mice was partially rescued in the rosiglitazone treatment group (Fig. 8B), while no change in gWAT mass was observed (Fig. 8C). Histologically, sWAT of rosiglitazone-treated ASKO mice showed smaller and newly differentiated adipocytes, suggesting an increase in lipid deposition capacity in the ASKO mice (Fig. 8D). The expression of adipokines, adipogenesis, lipogenesis and lipolysis genes were observed to be increased in gWAT (Fig. 8E–G) and sWAT (Fig. 8H–J) from the rosiglitozone-treated ASKO mice, compared to those from non-treated ASKO mice. Collectively, our data indicated that fat loss in the ASKO mice could be partially rescued by rosiglitazone treatment.

## Discussion

In this study, we explored the function of adipocyte RNF20 in regulating whole-body fat and energy metabolism by studying adipocyte-specific *Rnf20* knockout mice. These ASKO mice manifest dramatic and progressive fat loss, organomegaly and hyperinsulinemia, suggesting the impaired adipose tissue development and the consequence of metabolic disturbance.

In models of classical lipodystrophy, such as global *Agpat2*-knockout mice (Vogel et al., 2011) or adipocyte-specific *Bscl2*-knockout mice (Liu et al., 2014), the body weights are usually lower than control littermate despite organomegaly. It is of interest to note that CD-fed ASKO mice exhibited the greater body weight until 12 months of age. The body weight was determined majorly by the lean mass and fat mass. Despite of the slightly decreased fat mass, the significantly increased lean mass might contribute to the greater body weight of 6-month-old ASKO mice (Fig. 1B). When both groups of mice were aged to 12 months old, age-related fat deposition in the WT mice is similar to the increased lean mass in the ASKO mice (Fig. 1C), resulting in the similar body weight of both groups of mice. Similarly, this kind of change in body weight was accelerated under HFD condition. It looks like RNF20 deletion in adipocyte stimulates the lean mass growth and ASKO mice might be the good model to further investigate the crosstalk of adipose tissues and skeletal muscles.

Severe fat loss in lipodystrophic mouse models is companied by the ectopic fat deposition and development of metabolic conditions, such as extreme insulin resistance, diabetes and/or hepatic steatosis. Normal histological staining of the liver and non-significant changes of TAG in the liver of ASKO mice suggested that organomegaly was not due to ectopic fat deposition. This finding is in agreement with the observation from CD-fed *Rnf20*^+/−^ mice, which had lower fat content but no ectopic fat deposition (Jeon et al., 2020). Interestingly, despite the normal levels of hepatic TG, the slight but significantly increased serum TG was observed in the 6-month-old ASKO mice. Consider the reduced lipogenesis related gene expression, we suspected the defective lipid storage in WAT of ASKO mice might be the reason of increasing the circulating TG.

Surprisingly, we find that the ability of the ASKO mice to clear exogenous glucose was better than that exhibited by the WT mice. Coincidentally, similar ameliorated glucose tolerance was observed in CD-fed *Rnf20*^+/−^ mice (Jeon et al., 2020), HFD-fed *Pparγ*^+/−^ mice (Kubota et al., 1999), mice with compromised BAT lysine-specific demethylase1 (*Lsd1*) (Duteil et al., 2016) and mice without adipose insulin and IGF-1 signaling (Boucher et al., 2012). It has been reported that adipocyte hypertrophy and inflammation are closely related to insulin sensitivity and glucose metabolism (Jernas et al., 2006; Kim et al., 2015). The significantly induced the inflammation marker expressions rules out reduced inflammation in fat tissue as a cause for the improved glucose tolerance in the ASKO mice, but the smaller adipocytes of ASKO mice may be a contributing factor. In addition, the significant upregulation of *Glut4* in gastrocnemius of ASKO mice (Fig. S6), suggesting the increased glucose uptake in skeletal muscle. And coupled with the significant increased insulin levels in serum of ASKO mice (Fig. 3B), it seems likely that better glucose tolerance of ASKO mice is due to increased glucose uptake in skeletal muscle and increased insulin secretion.

Consistent with the phenotypes of WAT loss and BAT whitening in ASKO mice, it is of interest to find the complete opposite regulation of genes involved in adipogenesis, including CEBPα and PPARγ, in both tissues. These data suggested the distinct functions of RNF20 in WAT and BAT. Actually, the classical WAT and BAT have different developmental origin and lineage, therefore the genetic and epigenetic regulations of genes in both tissues are different, which may result in the distinct expression pattern of CEBPα and PPARγ in WAT and BAT from ASKO mice. Moreover, RNF20 has been reported to function as a E3 ubiquitin ligase for AP-2α and increases CEBPα through stimulating the polyubiquitination and proteasome-dependent degradation of AP-2α (Ren et al., 2014). Recently, another target of RNF20, nuclear corepressor 1 (NCOR1), was identified in preadipocyte 3T3-L1 cell, in which, RNF20 stimulates the transcriptional activity of PPARγ via promoting the proteasomal degradation of NCoR1 (Jeon et al., 2020). However, how RNF20 regulates the BAT program is still unknown and its potential ubiquitin targets in BAT have not been reported yet so far. These critical questions need to be addressed further with both ASKO and the BAT-specific *Rnf20* knockout mice in the future.

Note that the opposite expression pattern of BAT selective genes, including *Ucp1* and *Cox8b*, was also observed in WAT and BAT from ASKO mice, further suggesting the distinct transcriptional response of both tissues upon RNF20 depletion. The decreased UCP1 and OXPHOS protein expression in the BAT led to the extreme cold sensitivity of ASKO mice. It has been reported that the promoter region of *Ucp1* contains H3K4me3 and is activated in brown adipocytes (Pan et al., 2015) and H2Bub is the prerequisite of the H3K4me3 (Worden and Wolberger 2019). We checked the levels of H3K4me3 in BAT of WT and ASKO mice, as expected, decreased H3K4me3 levels were observed in the BAT of ASKO mice (Fig. S7), suggesting the epigenetic regulation of RNF20 on UCP1. In addition to Ucp1, two key transcriptional factors involved in the BAT biogenesis, PRDM16 and PGC1α, were also observed to be suppressed in BAT from ASKO mice (Fig. 6F). All these evidences suggesting the critical role of RNF20 as a key epigenetic regulator required regulating gene transcription to promote BAT biogenesis and function. The role of RNF20-mediated H2Bub in modulating chromatin structures has been reported in studies on meiotic recombination (Xu et al., 2016). We predicted that chromatin structure was changed in adipose tissues of ASKO mice, although this supposition needs to be further studied. The upregulation of the UCP1 and beige markers in the gWAT of the aged ASKO mice indicated WAT browning, although apparent beige-like adipocytes were not observed, suggesting that the WAT browning might be a compensation for the impaired BAT function.

Numerous studies have shown that in a disease state, organisms deposit less fat and have similar pathological features as that manifested by impaired lipogenesis (triglyceride synthesis and storage) and blocked adipogenesis (differentiation of preadipocytes to adipocytes) (Savage 2009). In agreement with this evidence, our *in vitro* data illustrated its critical role in adipocyte differentiation at both early and late stages. Rosiglitazone treatment could rescue the fat deposition in the ASKO mice. Consistent with the observation from rosiglitazone-treated adipose-specific *Seipin* knockout mice (Liu et al., 2014) and troglitazone-treated lipodystrophy patients (Arioglu et al., 2000), sWAT in our study showed a significant response to a PPARγ agonist. Collectively, our data suggested that *Rnf20* affected fat metabolism, at least partially, through the activity of PPARγ. The identification novel targetable molecules and signaling pathways of adipose tissue dysfunction and its related metabolic diseases will be advanced by these findings.

## Conclusions

In summary, our results showed that RNF20 plays an essential role in the regulation of adipocyte differentiation and adipose tissue function. Moreover, the finding that rosiglitazone treatment dramatically increases the sWAT of ASKO mice supports the notion that defective PPARγ accounts for the phenotype of our mice model. Modulating *Rnf20* regulated signaling cascades may contribute a potential target for the therapeutic invention of adipose tissue dysfunction and related metabolic diseases.

## Materials and Methods

### Animals and ethical statement

The mice used in the study had a C57BL/6 background. Homozygous *Rnf20*^*Flox*/*Flox*^ mice were obtained as previously described (Xu et al., 2016). The *Rnf20*^*Flox*/*Flox*^ mice were crossed with transgenic mice expressing Cre recombinase under the control of the *adiponectin* promoter to generate *Rnf20*^*Flox*/+^*adiponectin-Cre*^+^ mice, and the F1 generation was crossed with *Rnf20*^*Flox*/*Flox*^ mice to generate *Rnf20*^*Flox*/*Flox*^*adiponectin-Cre*^+^ (referred to as ASKO) mice, littermates *Rnf20*^*Flox*/*Flox*^*adiponectin-Cre*^−^ mice were used as the wild type control. For genotyping, genomic DNA extracted from mouse tails was subjected to PCR using the primers specific for *Cre* and *Rnf20* genes (Table S1). Male mice were studied in all cases. The PCR conditions were as follows: 95 °C for 3 min, 32 rounds of 94 °C for 30 s, 58 °C for 30 s, and 72 °C for 30 s, followed by 72 °C for 5 min. For high-fat diet (HFD) treatment, the HFD (Research Diet, D12492) was fed to mice weighing at least 20 g for 15 weeks. For rosiglitazone treatment, rosiglitazone (Sigma, St. Louis, MO)-containing chow diet (0.3 mg/g diet) was fed to 3-month-old mice for 10 weeks. All animal experiments were approved by the Animal Research Panel of the Committee on Research Practice of the University of the Chinese Academy of Sciences.

### Glucose and insulin tolerance tests

For the glucose tolerance tests (GTT), WT and ASKO mice were fasted overnight for 16 h with free access to drinking water. The glucose level was assessed following intraperitoneal injection of glucose (2 g per kg body mass). Tail vein blood glucose levels were determined immediately before and 30, 60, 90 and 120 min after glucose injection with a glucose analyzer (One Touch Ultra Easy, Shanghai). For the insulin tolerance tests (ITT), the WT and ASKO mice were fasted for 4 h with free access to drinking water. The glucose level was assessed following intraperitoneal injection of human insulin (0.5 U per kg body mass). The tail vein blood glucose levels were determined immediately before and 30, 60 and 90 min after insulin injection.

### Body composition, metabolic cage analysis and activity measurements

Fat and lean masses of the mice were measured by EchoMRI-700^TM^ (Echo MRI, USA). Oxygen consumption was measured for two consecutive days using a TSE lab master system. Briefly, the mice were placed in the measurement cages 4 h prior to data recording. The room temperature was maintained at 23 °C, and light/dark cycles as maintained in 12 h cycles. The volume of O_2_ consumed (VO_2_) were recorded every 12 min. The ambulatory activity of each mouse was measured using the optical beam technique (Opto-M3  Columbus Instruments, Columbus, OH, USA) over a period of two days and is expressed as 24-h average activity.

### Serum measurements

Serum was obtained by centrifugation of clotted blood, snap-frozen in liquid nitrogen and stored at −80 °C. The TG levels in serum and liver were determined using a commercially available assay kit (Applygen Technologies Inc., China). The levels of adiponectin and leptin in serum were determined using ELISA kits (Millipore, Billerica, MA, USA). All of these assays were performed according to the manufacturer’s instructions.

### Microscopy analysis

The mice were sacrificed, and the tissues were rapidly fixed in 4% PFA at 4 °C overnight, dehydrated, and embedded in paraffin for sectioning. The sections were stained with hematoxylin and eosin (H&E). Adipocyte size was measured using ImageJ version 1.46r (National Institutes of Health, USA). A total number of 250 adipocytes were measured for each fat depot for each mouse (three mice per group). For TEM, the adipose tissues were fixed in 2% (*v*/*v*) glutaraldehyde in 100 mmol/L phosphate buffer, pH 7.2, for 12 h at 4 °C. The samples were then postfixed in 1% osmium tetroxide, dehydrated in ascending gradations of ethanol, and embedded in fresh epoxy resin 618. Ultrathin sections (60–80 nm) were cut and stained with lead citrate before being examined with a Phillip CM-120 transmission electron microscope.

### Cold treatment

A cold tolerance test was performed with 2- and 6-month-old mice, which were placed in a cold chamber (4 °C) for as long as 3 h with free access to food and water, and their body temperature was measured using a rectal probe connected to a digital thermometer (Yellow Spring Instruments) every 1 h. Infrared pictures were taken with an infrared camera (FLIR Systems, Inc.).

### Cell culture and differentiation

The 3T3-L1 cells were cultured in DMEM/F12 containing 10% FBS and 1% penicillin/streptomycin. To determine differentiation levels, 2 days after reaching confluence, the cells were treated with culture medium supplemented with 5 μg/mL insulin, 0.5 mmol/L 3-isobutyl-1-methylxanthine, 1 μmol/L dexamethasone. After 2 days, the cells were transferred to culture medium supplemented with 5 μg/mL insulin and 1 μmol/L rosiglitazone for 2 days and then maintained in culture medium for another 4 days.

### RNAi experiments

Small interfering RNA (siRNA) transfections were carried out in 3T3-L1 cells with specific siRNAs targeting *Rnf20* gene and nonspecific control using lipofectamine™ RNAiMAX transfection reagent (Invitrogen) according to the manufacturer's protocol. The siRNA sequences are listed in Table S2.

### Oil red O staining

Differentiated adipocytes were stained with oil red O to verify the existence of lipid droplets. Briefly, the cells were washed twice with DPBS and fixed in 4% PFA for 10 min at room temperature and washed twice with distilled water. Then, 60% isopropanol was used to wash the cells. The cells were then stained with freshly diluted oil red O for 15 min. After staining, the cells were washed with distilled water and photographed using a microscope (Nikon, Japan).

### Quantitative real-time PCR

Total RNA was isolated using TRIzol reagent (Invitrogen) according to the manufacturer’s protocol, and cDNA was synthesized with a RevertAid First Strand cDNA synthesis kit (Thermo). Quantitative real-time PCR was performed in duplicate using SYBR Green PCR Master Mix on an ABI 7500 detection system (Applied Biosystems) with reaction volumes of 20 μL. All primer sequences are listed in Table S1. All PCR samples were quantitated by the comparative CT method to obtain relative quantitation that was normalized to *18S*.

### RNA-sequencing and gene ontology analysis

BAT from 6-month-old WT and ASKO mice were collected and the RNA samples was isolated. RNA-Sequencing, differentially expressed gene screening and Gene Ontology analyses were conducted as previously described (Tao et al., 2019).

### Lipidomic analysis

BAT from 6-month-old WT and ASKO mice were used for lipidomic analysis. The lipid extraction and mass spectrometric analyses were performed as described previously (Pan et al., 2019).

### Protein extraction and Western blot

The frozen mouse tissues were homogenized in tissue protein extraction reagent (Thermo 78510) supplemented with a protease and phosphatase inhibitor mini tablet (Thermo 88668) per 10 mL and then centrifuged for 20 min at 12,000 ×*g* to remove cell debris. The total protein concentrations were determined using a BCA kit (Thermo 23225). The proteins were separated on a 7.5%–12.5% SDS-PAGE gel and transferred to nitrocellulose membranes (Merck-Millipore). The membranes were blocked for one hour in TBST containing 5% skim milk and subsequently probed with primary antibodies overnight at 4 °C. Antibodies against β-TUBULIN (CST, 2146), RNF20 (Proteintech, 21625-1-AP), H2Bub (CST, 5546), H3K4me3 (abcam, ab8580), ATGL (CST, 2138), HSL (CST, 4107), pHSL (Ser660) (CST, 4126), pHSL (Ser565) (CST, 4137), pHSL (Ser563) (CST, 4139), PPARγ (CST, 2443), CEBPα (abcam, ab40764), UCP1 (abcam, ab10983), PRDM16 (abcam, ab106410), PGC1α (abcam, ab54481), Phospho-CEBPβ (Thr235) (CST, 3084), CEBPβ (Santa cruz, sc-7962), CYCLIND1 (CST, 55506S) and PCNA (CST, 2586) were used for western blot analysis. The blots were developed using anti-rabbit IgG, HRP-linked secondary antibody (CST, 7074) or anti-mouse IgG, HRP-linked secondary antibody (CST, 7076) and measured with an ECL-Plus system.

### Statistics

All statistical analyses were performed in GraphPad Prism Version 6 (http://www.graphpad.com/scientific-software/prism). Statistical comparisons between two groups were made using the two-tailed Student’s *t* test; comparisons between three or more groups were made by two-way ANOVA, followed by a Tukey’s *post hoc* test. All results are presented as the mean ± s.e.m. In all cases, differences were considered significant at *P < 0.05. *P*-values are indicated in each figure as *P < 0.05, **P < 0.01, or ***P < 0.001.

## Electronic supplementary material

Below is the link to the electronic supplementary material.

Supplementary material 1 (PDF 727 kb)

## References

[CR1] Arioglu E, Duncan-Morin J, Sebring N, Rother KI, Gottlieb N, Lieberman J, Herion D, Kleiner DE, Reynolds J, Premkumar A (2000). Efficacy and safety of troglitazone in the treatment of lipodystrophy syndromes. Ann Intern Med.

[CR2] Boucher J, Mori MA, Lee KY, Smyth G, Liew CW, Macotela Y, Rourk M, Bluher M, Russell SJ, Kahn CR (2012). Impaired thermogenesis and adipose tissue development in mice with fat-specific disruption of insulin and IGF-1 signalling. Nat Commun.

[CR3] Dawson MA, Kouzarides T, Huntly BJ (2012). Targeting epigenetic readers in cancer. N Engl J Med.

[CR4] Duan Y, Huo D, Gao J, Wu H, Ye Z, Liu Z, Zhang K, Shan L, Zhou X, Wang Y (2016). Ubiquitin ligase RNF20/40 facilitates spindle assembly and promotes breast carcinogenesis through stabilizing motor protein Eg5. Nat Commun.

[CR5] Duteil D, Tosic M, Lausecker F, Nenseth HZ, Muller JM, Urban S, Willmann D, Petroll K, Messaddeq N, Arrigoni L (2016). Lsd1 ablation triggers metabolic reprogramming of brown adipose tissue. Cell Rep.

[CR6] Fierz B, Chatterjee C, Mcginty RK, Bar-Dagan M, Raleigh DP, Muir TW (2011). Histone H2B ubiquitylation disrupts local and higher-order chromatin compaction. Nat Chem Biol.

[CR7] Fuchs G, Oren M (2014). Writing and reading H2B monoubiquitylation. Biochim Biophys Acta.

[CR8] Fuchs G, Shema E, Vesterman R, Kotler E, Wolchinsky Z, Wilder S, Golomb L, Pribluda A, Zhang F, Haj-Yahya M (2012). RNF20 and USP44 regulate stem cell differentiation by modulating H2B monoubiquitylation. Mol Cell.

[CR9] Himms-Hagen J, Melnyk A, Zingaretti MC, Ceresi E, Barbatelli G, Cinti S (2000). Multilocular fat cells in WAT of CL-316243-treated rats derive directly from white adipocytes. Am J Physiol Cell Physiol.

[CR10] Jeon YG, Lee JH, Ji Y, Sohn JH, Lee D, Kim DW, Yoon SG, Shin KC, Park J, Seong JK (2020). RNF20 functions as a transcriptional coactivator for PPARgamma by promoting NCoR1 degradation in adipocytes. Diabetes.

[CR11] Jernas M, Palming J, Sjoholm K, Jennische E, Svensson PA, Gabrielsson BG, Levin M, Sjogren A, Rudemo M, Lystig TC (2006). Separation of human adipocytes by size: hypertrophic fat cells display distinct gene expression. FASEB J.

[CR12] Jin W, Patti ME (2009). Genetic determinants and molecular pathways in the pathogenesis of Type 2 diabetes. Clin Sci (Lond).

[CR13] Karpiuk O, Najafova Z, Kramer F, Hennion M, Galonska C, Konig A, Snaidero N, Vogel T, Shchebet A, Begus-Nahrmann Y (2012). The histone H2B monoubiquitination regulatory pathway is required for differentiation of multipotent stem cells. Mol Cell.

[CR14] Kim JI, Huh JY, Sohn JH, Choe SS, Lee YS, Lim CY, Jo A, Park SB, Han W, Kim JB (2015). Lipid-overloaded enlarged adipocytes provoke insulin resistance independent of inflammation. Mol Cell Biol.

[CR15] Kouzarides T (2007). Chromatin modifications and their function. Cell.

[CR16] Kubota N, Terauchi Y, Miki H, Tamemoto H, Yamauchi T, Komeda K, Satoh S, Nakano R, Ishii C, Sugiyama T (1999). PPAR gamma mediates high-fat diet-induced adipocyte hypertrophy and insulin resistance. Mol Cell.

[CR17] Lee JH, Lee GY, Jang H, Choe SS, Koo SH, Kim JB (2014). Ring finger protein20 regulates hepatic lipid metabolism through protein kinase A-dependent sterol regulatory element binding protein1c degradation. Hepatology.

[CR18] Liang Q, Xia W, Li W, Jiao J (2018). RNF20 controls astrocytic differentiation through epigenetic regulation of STAT3 in the developing brain. Cell Death Differ.

[CR19] Liang X, Pan J, Cao C, Zhang L, Zhao Y, Fan Y, Li K, Tao C, Wang Y (2019). Transcriptional response of subcutaneous white adipose tissue to acute cold exposure in mice. Int J Mol Sci.

[CR20] Liu L, Jiang Q, Wang X, Zhang Y, Lin RC, Lam SM, Shui G, Zhou L, Li P, Wang Y (2014). Adipose-specific knockout of SEIPIN/BSCL2 results in progressive lipodystrophy. Diabetes.

[CR21] Minsky N, Shema E, Field Y, Schuster M, Segal E, Oren M (2008). Monoubiquitinated H2B is associated with the transcribed region of highly expressed genes in human cells. Nat Cell Biol.

[CR22] Nakamura K, Kato A, Kobayashi J, Yanagihara H, Sakamoto S, Oliveira DV, Shimada M, Tauchi H, Suzuki H, Tashiro S (2011). Regulation of homologous recombination by RNF20-dependent H2B ubiquitination. Mol Cell.

[CR23] Oelkrug R, Polymeropoulos ET, Jastroch M (2015). Brown adipose tissue: physiological function and evolutionary significance. J Comp Physiol B.

[CR24] Pan D, Huang L, Zhu LJ, Zou T, Ou J, Zhou W, Wang YX (2015). Jmjd3-mediated H3K27me3 dynamics orchestrate brown fat development and regulate white fat plasticity. Dev Cell.

[CR25] Pan J, Tao C, Cao C, Zheng Q, Lam SM, Shui G, Liu X, Li K, Zhao J, Wang Y (2019). Adipose lipidomics and RNA-Seq analysis revealed the enhanced mitochondrial function in UCP1 knock-in pigs. Biochim Biophys Acta Mol Cell Biol Lipids.

[CR26] Phillips KJ (2019). Beige fat, adaptive thermogenesis, and its regulation by exercise and thyroid hormone. Biology (Basel).

[CR27] Ren P, Sheng Z, Wang Y, Yi X, Zhou Q, Zhou J, Xiang S, Hu X, Zhang J (2014). RNF20 promotes the polyubiquitination and proteasome-dependent degradation of AP-2alpha protein. Acta Biochim Biophys Sin (Shanghai).

[CR28] Savage DB (2009). Mouse models of inherited lipodystrophy. Dis Model Mech.

[CR29] Sethi G, Shanmugam MK, Arfuso F, Kumar AP (2018). Role of RNF20 in cancer development and progression - a comprehensive review. Biosci Rep.

[CR30] So CC, Ramachandran S, Martin A (2019). E3 ubiquitin ligases RNF20 and RNF40 are required for double-stranded break (DSB) repair: evidence for monoubiquitination of histone H2B lysine 120 as a novel axis of DSB signaling and repair. Mol Cell Biol.

[CR31] Tao C, Wang Y, Zhao Y, Pan J, Fan Y, Liang X, Cao C, Zhao J, Petris MJ, Li K (2019). Adipocyte-specific disruption of ATPase copper transporting alpha in mice accelerates lipoatrophy. Diabetologia.

[CR32] Tarcic O, Pateras IS, Cooks T, Shema E, Kanterman J, Ashkenazi H, Boocholez H, Hubert A, Rotkopf R, Baniyash M (2016). RNF20 links histone H2B ubiquitylation with inflammation and inflammation-associated cancer. Cell Rep.

[CR33] Tseng YH, Cypess AM, Kahn CR (2010). Cellular bioenergetics as a target for obesity therapy. Nat Rev Drug Discov.

[CR34] Van Marken Lichtenbelt W D, Vanhommerig JW, Smulders NM, Drossaerts JM, Kemerink GJ, Bouvy ND, Schrauwen P, Teule GJ (2009). Cold-activated brown adipose tissue in healthy men. N Engl J Med.

[CR35] Vethantham V, Yang Y, Bowman C, Asp P, Lee JH, Skalnik DG, Dynlacht BD (2012). Dynamic loss of H2B ubiquitylation without corresponding changes in H3K4 trimethylation during myogenic differentiation. Mol Cell Biol.

[CR36] Virtanen KA, Lidell ME, Orava J, Heglind M, Westergren R, Niemi T, Taittonen M, Laine J, Savisto NJ, Enerback S (2009). Functional brown adipose tissue in healthy adults. N Engl J Med.

[CR37] Vogel P, Read R, Hansen G, Wingert J, Dacosta CM, Buhring LM, Shadoan M (2011). Pathology of congenital generalized lipodystrophy in Agpat2−/− mice. Vet Pathol.

[CR38] Woo Park J, Kim KB, Kim JY, Chae YC, Jeong OS, Seo SB (2015). RE-IIBP Methylates H3K79 and Induces MEIS1-mediated Apoptosis via H2BK120 Ubiquitination by RNF20. Sci Rep.

[CR39] Worden EJ, Wolberger C (2019). Activation and regulation of H2B-Ubiquitin-dependent histone methyltransferases. Curr Opin Struct Biol.

[CR40] Wu C, Cui Y, Liu X, Zhang F, Lu LY, Yu X (2020). The RNF20/40 complex regulates p53-dependent gene transcription and mRNA splicing. J Mol Cell Biol.

[CR41] Xu Z, Song Z, Li G, Tu H, Liu W, Liu Y, Wang P, Wang Y, Cui X, Liu C (2016). H2B ubiquitination regulates meiotic recombination by promoting chromatin relaxation. Nucleic Acids Res.

[CR42] Zingaretti MC, Crosta F, Vitali A, Guerrieri M, Frontini A, Cannon B, Nedergaard J, Cinti S (2009). The presence of UCP1 demonstrates that metabolically active adipose tissue in the neck of adult humans truly represents brown adipose tissue. FASEB J.

