# Supplementary material for: Rnf20 deficiency in adipocyte impairs adipose tissue development and thermogenesis
Source: Protein Cell. 2020 Aug 14;12(6):475–92. doi: 10.1007/s13238-020-00770-2 (PMC8160045; doi:10.1007/s13238-020-00770-2)
Supplement: Supplementary file 1 — Supplementary material 1 (PDF 727 kb) [file 13238_2020_770_MOESM1_ESM.pdf]

Supplementary materials

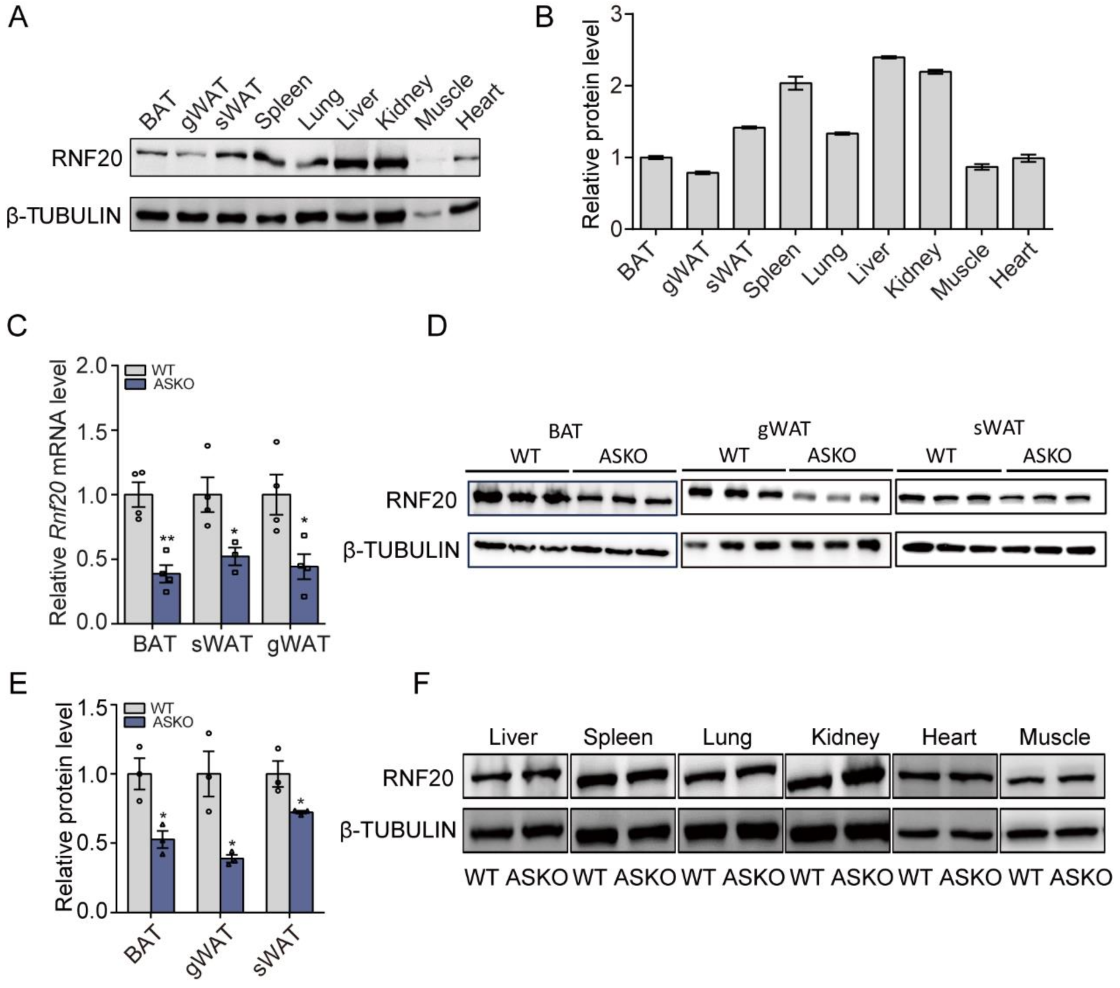

Supplementary Figure 1. Expression profile of RNF20 and the validation of adipocyte-specific *Rnf20* knockout mice. (A and B) Tissue distribution of RNF20 in 2-month-old male C57BL/6 mice (A) and the quantitative data (B). (C) *Rnf20* expression in the BAT, gWAT and sWAT from 2-month-old WT and ASKO mice (n = 4). (D and E) Protein expression of RNF20 in adipose tissues of 2-month-old WT and ASKO mice, as determined by immunoblot analysis (D) and the quantitative analysis. (F) RNF20 expression in various tissues, including liver, spleen, lung, kidney, heart and muscle in the 2-month-old WT and ASKO mice. Note that the levels of RNF20 expression were not changed in these tissues. Data are presented as the mean ± s.e.m. $*p < 0.05$, $**p < 0.01$.

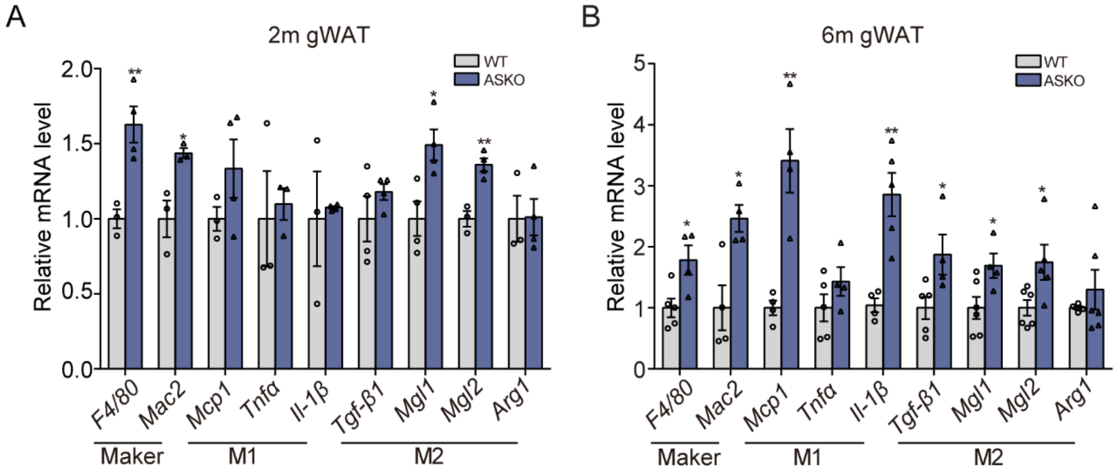

Supplementary Figure 2. Relative mRNA levels of macrophage markers and inflammatory cytokines in gWAT from 2-(A), and 6-month-old (B) WT and ASKO mice (n = 4 - 6). Data are presented as the mean ± s.e.m.*p < 0.05, **p < 0.01 and ***p < 0.001 for differences between WT and ASKO mice.

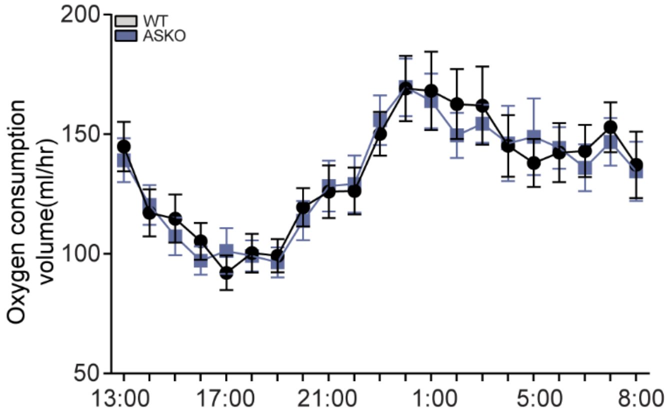

Supplementary Figure 3. The whole-body oxygen consumption rate (VO_2) of 6-month-old WT and ASKO mice (n = 6). Data are presented as the mean ± s.e.m.

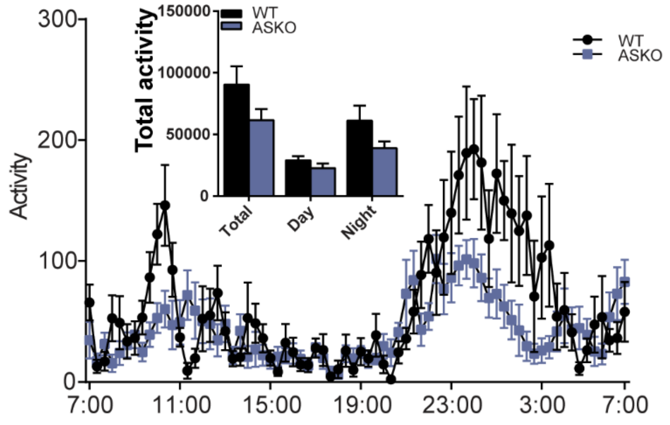

Supplementary Figure 4. Physical activity were measured with the 6-month-old WT and ASKO mice throughout the day and night (n = 8). Data are presented as the mean ±s.e.m.

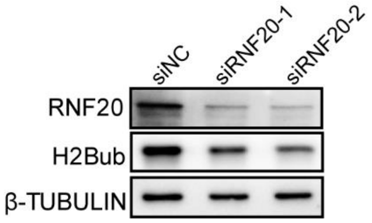

Supplementary Figure 5. The siRNAs efficiency test in 3T3-L1 cells.

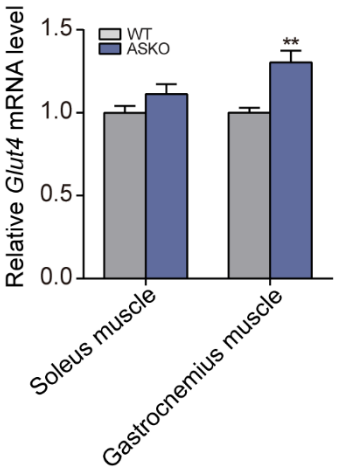

Supplementary Figure 6. Relative *Glut4* mRNA level in soleus muscle and gastrocnemius muscle from 9-month-old mice. Data are presented as the mean ±s.e.m. $^{**}p < 0.01$ for differences between WT and ASKO mice.

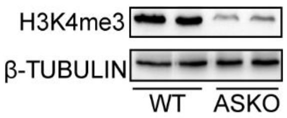

Supplementary Figure 7. Representative Western blot of H3K4me3 in BAT of the 6-month-old WT and ASKO mice.

Supplementary table 1. Primers used in this study

Name	Sequence 5′-3′
Rnf20-genotyping-F	GCTGTAAGAGTTCTTAATGTATG
Rnf20-genotyping-R	GGCTTGTCACACAAGCATGAGCATC
Cre-F	GCCTGCATTACCGGTCGATGC
Cre-R	CAGGGTGTTATAAGCAATCCC
18s-F	GTAACCCGTTGAACCCCATT
18s-R	CCATCCAATCGGTAGTAGCG
Rnf20-qPCR-F	GTGAACCGGTACTGGAGCCAG
Rnf20-qPCR-R	GAAAGCTGGCTCTTGCCCATC
Cebpα-qPCR-F	CCCTTGCTTTTTGCACCTCC
Cebpα-qPCR-R	GCTTTCTGGTCTGACTGGGG
Pparγ-qPCR-F	CGGAAGCCCTTTGGTGACTT
Pparγ-qPCR-R	CCTCGATGGGCTTCACGTTC
Srebp1c-qPCR-F	GGAGCCATGGATTGCACATT
Srebp1c-qPCR-R	GCTTCCAGAGAGGAGGCCAG
Acc-qPCR-F	GAGAGGGGTCAAGTCCTTCC
Acc-qPCR-R	ACATCCACTTCCACACACGA
Dgat1-qPCR-F	ATCTGAGGTGCCATCGTC
Dgat1-qPCR-R	ATGCCATACTTGATAAGGTTCT
Dgat2-qPCR-F	TCAACCGAGACACCATAGAC
Dgat2-qPCR-R	CCTCAAAGATCACCTGCTT
Fas-qPCR-F	GGGTCTATGCCACGATTC
Fas-qPCR-R	GTGTCCCATGTTGGATTTG
Scd1-qPCR-F	CTGACCTGAAAGCCGAGAAG
Scd1-qPCR-R	GCGTTGAGCACCAGAGTGTA
Fabp4-qPCR-F	TCACCTGGAAGACAGCTCCT
Fabp4-qPCR-R	AATCCCCATTTACGCTGATG
Adiponectin-qPCR-F	CTCCTGCTTTGGTCCCTC
Adiponectin-qPCR-R	GCCAGTGCTGCCGTCATA
Resistin-qPCR-F	TCCTTGTCCCTGAACTGC
Resistin-qPCR-R	ACGAATGTCCCACGAGCC
Leptin-qPCR-F	CACAGTCTGGAGCGAAGG
Leptin-qPCR-R	CACAATCTGGGAACAAGC
Ucp1-qPCR-F	GTGAAGGTCAGAATGCAAGC
Ucp1-qPCR-R	AGGGCCCCCTTCATGAGGTC
Mcp1-qPCR-F	GTTAACGCCCCACTCACCTG
Mcp1-qPCR-R	CCTTCTTGGGGTCAGCACAG
Tnfa-qPCR-F	AATGGCCTCCCTCTCATCAG
Tnfa-qPCR-R	CCCTTGAAGAGAACCTGGGA
Hsl-qPCR-F	GACTCACCGCTGACTTCC
Hsl-qPCR-R	TGTCTCGTTGCGTTTGTAG
Atgl-qPCR-F	CGTCACCTGTGCCTTACTC
Atgl-qPCR-R	GGGCTCAAACTCCCAAAC

Cpt1α-qPCR-F	CCAGGCTACAGTGGGACATT
Cpt1α-qPCR-R	GAACTTGCCCATGTCCTTGT
Cpt2-qPCR-F	TATGATGGCTGAGTGCTCCA
Cpt2-qPCR-R	ATAGAGCTCAGGCAGGGTGA
Srebp2-qPCR-F	CCCTTGACTTCCTTGCTGCA
Srebp2-qPCR-R	GCGTGAGTGTGGGCGAATC
Lxrα-qPCR-F	ATGTCCACGAGTGACTGTT
Lxrα-qPCR-R	TTGACTCTCCCTTAATGCTAC
Lxrβ-qPCR-F	TATGCCTTGCTTATCGCCATC
Lxrβ-qPCR-R	CTTGTCCTGGAGTCGCAATG
Cox4-qPCR-F	CGGCGTGACTACCCCTTG
Cox4-qPCR-R	TGAGGGATGGGGCCATACA
Cycs-qPCR-F	CCAAATCTCCACGGTCTGTTC
Cycs-qPCR-R	ATCAGGGTATCCTCTCCCCAG
Acadl-qPCR-F	TTGGTGGGGACTTGCTCTCA
Acadl-qPCR-R	CTGTTCTTTTGTGCCGTAAT
Acadm-qPCR-F	GGATGACGGAGCAGCCAATG
Acadm-qPCR-R	ATACTCGTCACCCTTCTTCT
Crp-qPCR-F	ATGGAGAAGCTACTCTGGTGC
Crp-qPCR-R	ACACACAGTAAAGGTGTTCAGTG
Elovl6-qPCR-F	AAGCAGTTCAACGAGAACGAA
Elovl6-qPCR-R	CGTACAGCGCAGAAAACAGG
Prdm16-qPCR-F	TGAGCCCCAAGGAGTCTATG
Prdm16-qPCR-R	GTCGGCTCCAAAGCTAACAG
Adrb3-qPCR-F	TTTGATGGCTATGAAGGTGCG
Adrb3-qPCR-R	CGTTGCTTGTCTTTCTGGAGG
Cox6a1-qPCR-F	TGCTCAACGTGTTCCTCAAG
Cox6a1-qPCR-R	TAAGGGTCCAAAACCAGTGC
Slc27a2-qPCR-F	ATGCCGTGTCCGTCTTTTAC
Slc27a2-qPCR-R	CGATGATGATTGATGGTTGC
Cox8b-qPCR-F	GAACCATGAAGCCAACGACT
Cox8b-qPCR-R	GCGAAGTTCACAGTGGTTCC
Oplah-qPCR-F	CTTCACGCACGTCTCCTTGT
Oplah-qPCR-R	GCATCTGCACAGGCCGTAT
Cox17-qPCR-F	ATAGTTGCTTTCGCCTGGAA
Cox17-qPCR-R	ACAAAGTAGGCCACCACGTC
Apoe-qPCR-F	GGTTCGAGCCAATAGTGGAA
Apoe-qPCR-R	TATTAAGCAAGGGCCACCAG
Fads1-qPCR-F	AAGGCCAACCACCTCTTCTT
Fads1-qPCR-R	ACTGACAGGTGCCCAAAGTC
Pdk3-qPCR-F	TTTGAGAGGCTGCCAGTTTT
Pdk3-qPCR-R	CGTCTCTGGTTGACTTGCAG
Egfr-qPCR-F	ACACTGCTGGTGTTGCTGAC
Egfr-qPCR-R	CCCAAGGACCACTTCACAGT

Bmp4-qPCR-F	GCTGGAATGATTGGATTGTGG
Bmp4-qPCR-R	ATGGCATGGTTGGTTGAGTTG
Acox1-qPCR-F	CAGGAAGAGCAAGGAAGTGG
Acox1-qPCR-F	CCTTTCTGGCTGATCCCATA
Klhl13-qPCR-F	AGAATTGGTTGCTGCAATACTCC
Klhl13-qPCR-R	AAGGCACAGTTTCAAGTGCTG
Cd137-qPCR-F	CGTGCAGAACTCCTGTGATAAC
Cd137-qPCR-R	GTCCACCTATGCTGGAGAAGG
Cd40-qPCR-F	TTGTTGACAGCGGTCCATCTA
Cd40-qPCR-R	CCATCGTGGAGGTACTGTTTG
Ear2-qPCR-F	CCTGTAACCCCAGAACTCCA
Ear2-qPCR-R	CAGATGAGCAAAGGTGCAAA
Sp100-qPCR-F	TGATGGAGGGAACCCAAACTC
Sp100-qPCR-R	CTTCCTTGAGAATAGCTGGCAC
Tbx1-qPCR-F	GGCAGGCAGACGAATGTTC
Tbx1-qPCR-R	TTGTCATCTACGGGCACAAAG
Tmem26-qPCR-F	ACCCTGTCATCCCACAGAG
Tmem26-qPCR-R	TGTTTGGTGGAGTCCTAAGGTC
F4/80-qPCR-F	TTTCCTCGCCTGCTTCTTC
F4/80-qPCR-R	CCCCGTCTCTGTATTCAACC
Mac2-qPCR-F	CGGGTGGAGCACTAATCAGG
Mac2-qPCR-R	GTAGGCCCCAGGATAAGCAG
Il-1β-qPCR-F	TGCCACCTTTTGACAGTGATG
Il-1β-qPCR-R	AAGGTCCACGGGAAAGACAC
Tgf-β1-qPCR-F	GGCGGTGCTCGCTTTGTA
Tgf-β1-qPCR-R	TCCCGAATGTCTGACGTATTGA
Mgl2-qPCR-F	GGATGGGACCGACTTTGA
Mgl2-qPCR-R	GTGGGCTGAGCTGGCTTT
Mgl1-qPCR-F	TGAGAAAGGCTTTAAGAACTGGG
Mgl1-qPCR-R	GACCACCTGTAGTGATGTGGG
Arg1-qPCR-F	AAGACAGCAGAGGAGGTG
Arg1-qPCR-R	AGTCAGTCCCTGGCTTAT

Supplementary table 2. List of siRNA used in this study

Gene	sense (5'-3')	antisense (5'-3')
siRNF20-1	GAGAUUCUGUUAAGGAUAATT	UUAUCCUUAACAGAAUCUCTT
siRNF20-2	GCGAAUCAAGUCUAAUCAGTT	CUGAUUAGACUUGAUUCGCTT
siNC	UUCUCCGAACGUGUCACGUTT	ACGUGACACGUUCGGAGAATT
